# Normal electrocardiographic and echocardiographic (M-mode and two-dimensional) values in Polish Landrace pigs

**DOI:** 10.1186/s13028-014-0054-2

**Published:** 2014-09-09

**Authors:** Urszula Paslawska, Agnieszka Noszczyk-Nowak, Robert Paslawski, Adrian Janiszewski, Liliana Kiczak, Dorota Zysko, Jozef Nicpon, Ewa A Jankowska, Andrzej Szuba, Piotr Ponikowski

**Affiliations:** Regional Specialist Hospital in Wroclaw, Research and Development Centre, Kamienskiego Street 73a, 51-124 Wroclaw, Poland; Department of Internal and Diseases with Clinic for Horses, Dogs and Cats, Faculty of Veterinary Medicine, Wroclaw University of Environmental and Life Sciences, Grunwaldzki Sq. 47, 50-366 Wroclaw, Poland; Department and Clinic of Internal and Occupational Diseases and Hypertension, Wroclaw Medical University, Borowska Street 101, 50-556 Wroclaw, Poland; Department of Biochemistry, Pharmacology and Toxicology, Faculty of Veterinary Medicine, Wroclaw University of Environmental and Life Sciences, Norwida Street 25/27, 50-375 Wrocław, Poland; Department of Emergency Medicine, Wroclaw Medical University, Bartla Street, 51-618 Wrocław, Poland; Centre for Heart Diseases, Military Hospital, Weigla Street 5, 50-996 Wroclaw, Poland; Department of Heart Diseases, Wrocław Medical University, Weigla Street 5, 50-556 Wrocław, Poland

## Abstract

**Background:**

Swine are recognized animal models of human cardiovascular diseases. Normal values of cardiac morphology and function have been published for swine but for smaller number of pigs and not for swine whose weights ranged up 100 kg. In order to improve the value of results of an investigation on cardiac morphology and function in swine when such data are extrapolated to humans, the aim of this study was to document electrocardiographic and echocardiographic measures of cardiac morphology and function in swine. The study comprised 170 single and repeated measurements that were made in 132 healthy domestic swine (*Sus domesticus*) whose weights ranged between 20-160 kg and were used as controls in three different experiments. All electrocardiographic and echocardiographic measurements in all swine were done under general anaesthesia.

**Results:**

Statistically significant correlations were found between body weight and heart rate (HR), the duration of the P-wave, the duration of the QRS interval, the duration of the QT interval, and the corrected QT ratio (QTc). Since body weight was positively correlated with age, statistically significant correlations were also found between age and HR, the duration of the P-wave, the duration of the QRS interval, the duration of the QT interval, and the QTc. We found that the thickness of the left ventricular wall and the internal diameter of the left ventricle increased with age and body weight. We also found positive trends between body weight and ejection fraction and body weight and fractional shortening. We also found a positive relationship between age, body weight, and the ratio of the left ventricular internal diameter to its wall thickness, as well as the relative left atrial size.

**Conclusion:**

Many electro- and echocardiographic measures of cardiac morphology and function of healthy swine are related to their body weight. When the electro- and echocardiographic measures of domestic swine and humans are compared, the most comparable electrocardiographic values are those that were determined in swine whose body weights are not greater than 70 kg. In contrast, the most comparable echocardiographic measures are those that were determined in swine with a body weight of 40–110 kg.

## Background

Swine are recognized animal models of human cardiovascular diseases [1-4]. The use of swine in human cardiovascular research has progressively increased because of similarities in their heart sizes, the diameters of their coronary arteries, their propensity to spontaneously develop atherosclerosis, and their predisposition to sudden cardiac death (SCD) [1-3]. Although heart size is proportional to body weight, specific differences in this interdependence exist among animal species and breeds. Normal values of cardiac morphology and function have been published for dogs [5], cattle [6], ferrets [7], sheep [8], monkeys [9], Syrian hamsters [10], mice [11], and rats [12]. However, published information about cardiac morphology and function in swine is lacking. Specifically, the documented information on cardiac size and function in swine applies only to young swine up to the age of 112 days and a body weight of 40 kg and to minipigs [13,14]. In order to improve the value of results of an investigation on cardiac morphology and function in swine when such data are extrapolated to humans, the aim of this study was to document electrocardiographic and echocardiographic measures of cardiac morphology and function in swine whose weights ranged between 20–160 kg.

## Methods

The study comprised 170 single and repeated measurements that were made in 132 healthy Polish landrace pigs (65 boars and 67 sows) whose weights ranged between 20–160 kg and were used as controls in three different experiments. The repeated measurements were made in those pigs whose body weights increased by more than 10 kg during the study period (86 pigs were measured ones, 30 pigs were measured twice and 8 pigs were measured thrice. Minimum time span between measures were 1 month). Pigs were selected for inclusion in the investigation if they were found healthy (a) on clinical examination and (b) when their blood cell morphology, haematocrit determination, plasma haemoglobin levels, and serum creatinine, urea, total protein, albumin, and electrolyte levels were within the normal referenced range. All the animals were acclimated for two weeks before any measurements were made. All swine were singly housed in pens in a room at a room temperature of 18-20 °C and a relative humidity of 60-75%. The pens were cleaned twice daily. Swine were fed a diet (90.44% dry weight) which contained 14.7% protein, 3.1% fat, 4.7% crude protein, 6.06% ash, 0.5% salt (NaCl), 1.05% calcium, 0.77% phosphorus, 0.62% lysine, 0.24% methionine, 0.3% cysteine, 0.48% threonine, 0.183% tryptophan, 13243 IU/kg vitamin A, 2000 IU/kg vitamin D_3_, 81.65 mg/kg vitamin E, 4.11 mg/kg vitamin B_1_, 7.16 mg/kg vitamin B_2_, 50.22 mg/kg Niacin (vitamin B_3_), 24.29 mg/kg vitamin B_5_, 6.11 mg/kg vitamin B_6_, and 36 μg/kg vitamin B_12_. All study pigs had unlimited access to water. The swine were assigned to groups according to their body weight in 10 kg increments. All swine received humane care in compliance with the 8^th^ edition of the *Guide for the Care and Use of Laboratory Animals* which was published by the National Institutes of Health (http://grants.nih.gov/grants/olaw/Guide-for-the-care-and-use-of-laboratory-animals.pdf) [15]. The investigation was conducted in accordance with the guidelines for experimentation on animals of the bioethical committee of the Wrocław University of Environmental and Life Sciences. At the end of the investigation, the swine were returned to their original investigation and were humanely killed according to the requirements of the original investigation.

All measurements were collected under identical conditions of general anaesthesia with food restriction for 12 hours (the pigs were fed in the morning of the day before the examination and the food was removed in the afternoon) and water restriction for four hours before the anaesthesia. Since it is very difficult to perform cardiac studies on conscious pigs without immobilisation, we developed an immobilising protocol for measuring each pig’s cardiovascular and respiratory function by blood sampling, electrocardiography, and echocardiography. In this protocol, each pig is first premedicated by an intramuscular injection of a mixture of 0.1 mg/kg midazolam (Midanium, WZF Polfa S.A), 0.02 mg/kg medetomidine (Cepetor, CP-Pharma Handelsges), and 8 mg/kg ketamine (Bioketan, Vetoquinol, Biowet sp. z o.o.). The pig is then immobilised by general anaesthesia which is achieved by a single intravenous bolus injection of either sodium thiopental (1–5 mg/kg) (Thiopental, Sandoz) or propofol (1–2.5 mg/kg). (Propofol, MTC/LCT Fresenius Kabi). The duration of the immobilisation is as long as 60 minutes, during which blood sampling and reproducible electrocardiographic and echocardiographic measurements can be made. For collecting the electrocardiographic record and capturing the echocardiographic images, each pig was placed in the left lateral position with the front legs slightly stretched forward in a room whose temperature was set at 21°C. Once anaesthetised, the vital signs of each pig, namely tongue pulse oximetry, non-invasive blood pressure, respiration rate, and body temperature, were monitored by a LIFEPAK® 12 multiparameter monitor (Physio-Control, Inc., Redmond, WA, USA). All measurements were collected over 20–30 minutes once the animal was anaesthetised and heart function was stable.

The electrocardiogram (ECG) in each pig was recorded when the pig was in the left lateral position using an electrocardiograph with noise and muscle tremor filtering (Model BTL SD08, BTL Company, Czech Republic. The ECG signals were recorded as a direct electronic signal every 30 seconds using the electrocardiograph’s software. The electrodes were placed on the right forearm (RA), the left forearm (LA), the right leg (RL), and the left leg (LL) [14,16]. The electrodes for the V_1_, V_2_, and V_4_ precordial leads were placed to the right of sternum in the third intercostal space (V_1_), just to the left of the sternum (V_2_), and to the left at the costochondral junction in the fourth intercostal space (V_4_). According to Crick and colleagues [17], these locations correspond to the anatomical positions of the right ventricle, the interventricular septum, and the left ventricle. Each ECG was analysed to determine the heart rate (HR), the amplitude and duration of the P-wave, the duration of the PQ interval, the duration of the QRS interval, the amplitude of the R-wave, the duration of the QT interval, the corrected QT ratio (QTc), and the heart’s electrical axis in lead II, as measured by the voltage between the LL and RA electrodes. Although the swine were deemed as clinically healthy at the time of entry into the study, the output from all leads was used to confirm their cardiac health.

The echocardiographic measurements were done by one researcher over at least three consecutive cardiac cycles when each pig was in the left lateral position using an Aloka 4000+ echocardiograph (Aloka Company, Japan) and a 3.5 MHz transducer according to the guidelines of the American Society for Echocardiography. The following cardiac dimensions were determined from the echocardiograms: the relative left atrial size was estimated from the left atrial (LA)-to-aortic root (Ao) diameter ratio (LA/Ao). This measurement was determined from images that were collected from a right short-axis view at base of the heart. The probe was placed in the right third intercostal space over the sternum. The end-diastolic and end-systolic thickness of the interventricular septum (IVSd and IVSs) and left ventricle posterior wall (LWPd and LVPVs) was measured from images that were collected from a right long-axis four-chamber view (after moving the probe to caudal and rotated 90°). Estimates of left ventricular systolic function were obtained from the index of circumferential myocardial contraction and fractional shortening (FS) using the Teicholz formula ([LVIDd – LVIDs/LVIDd] x100), where LVIDd and LVIDs are the internal left ventricular dimensions at end-diastole and end-systole, respectively. Estimates of left ventricular end-diastolic volume (LVEDV), the end-systolic volume (LVESV), the stroke volume (LVSV), and ejection fraction (EF) were calculated by the echocardiograph’s software using the formula ([LVEDV – LVESV/LVEDV] x100). The following additional measures of systolic function were calculated: the ratio of end-systolic and end-diastolic left ventricular internal diameters to the thickness of the left ventricular walls at end-systole and end-diastole and the sum of maximum wall thickness values in end-diastole (interventricular septum in diastole (IVSd) and left ventricular posterior wall in end-diastole (LPWd), The left ventricular relative wall thickness (RWT) was calculated using the equation, 2 × LPWd/LVIDd. The percentage systolic thickening (ST) of the interventricular septum (IVS) was calculated using the equation, ST IVS% = (IVSs-IVSd/IVSs) × 100. The percentage systolic thickening of the left ventricular free wall was calculated using the equation, ST LPW% = (LPWs-LPWd/LPWs) × 100.

Left ventricular systolic and diastolic functions were also measured by pulsed-waved tissue Doppler ultrasonography. For this purpose, the Doppler gate was placed over the basal segment of the left ventricular free wall just below the mitral annulus in the right parasternal short-axis view and also in the apical four-chamber view for some swine. For this four-chamber view, the probe was placed in fourth left intercostal space over the sternum by sliding the probe under chest wall. For swine with a body weight greater than 130 kg, good quality images from the apical view were not obtained in 20-50% of the pigs: image quality progressively reduced as body fat and weight increased. From this reason, only measurements that obtained from images that were collected in the right parasternal short-axis view were used. Measurements of myocardial velocities during early systole (Sm1), systole (Sm2), early diastole or early filling (Em), and late diastole or atrial contraction (Am) were made and the Em/Am ratio was calculated in all pigs. The isovolumic relaxation time was measured from the onset of QRS complex to the onset of Sm2 wave and was named as RIVRT. The time of onset of the QRS complex to the peak of the Sm2 wave was also measured using tissue Doppler imaging (TDI) and was named as RIVRTp.

### Statistical analysis

A computerized software package (Statistica for Windows, version 8.0, StatSoft, Poland) was used to statistically analyse the data. The electrocardiographic and echocardiographic data, which were not normally distributed, were compared by Mann–Whitney U test and Spearman’s rank-order correlation coefficient was used for assessing the interdependence between the measured variables. Correlation was calculated only for one examination of one animal. The data from the second examination was excluded from analysis of correlation. All data are displayed as the mean ± standard deviation and statistical significance was set at 5%.

## Results

During the electro-and echocardiographic examinations, all monitored variables of the anaesthetised pigs were within their reference ranges.

**Figure 1:**
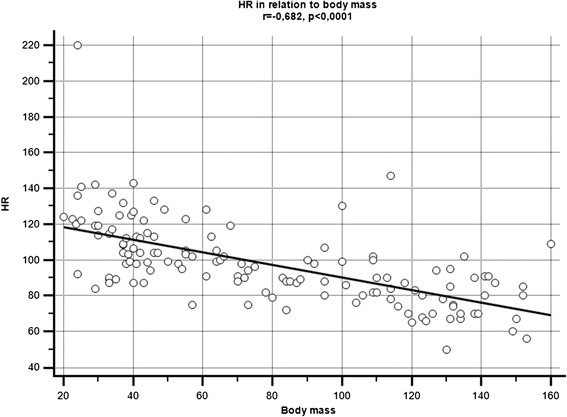
**The correlation between heart rate (bmp) and body weight (kg) in healthy domestic swine.** HR is displayed as beats/minute and body weight in kilograms. r = − 0.682; *P* < 0.0001.

**Figure 2:**
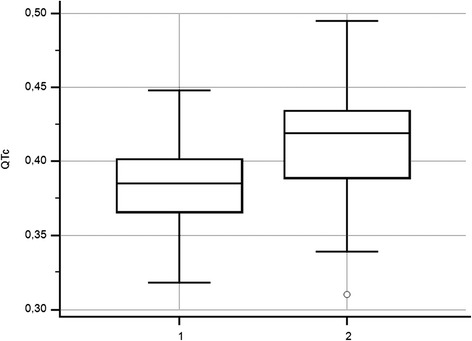
**The corrected QT intervals (QTc) (ms/√s) in healthy swine whose body weights are less than 70 kg.** Group 1; n = 68, QTc ratio = 0.386 ± 0.03 and body weight than 70 kg. Group 2; n = 64, QTc ratio = 0.0441 ± 0.04 and body weight between 70–160 kg.

The results of the ECG analysis of swine according to each weight group and age are presented in Tables 1 and 2. Statistically significant correlations (*P* < 0.05) were found between body weight and (a) HR (r = −0.682; Figure 1), (b) the duration of the P-wave (r = 0.47), (c) the duration of the QRS interval (r = 0.338), (d) the duration of the QT interval (r = 0.719), and the QTc ratio (r = 0.464). Since body weight was positively correlated (r = 0.92, *P* < 0.05) with age, statistically significant correlations (*P* < 0.05) were also found between age and (a) HR (r = −0.66), (b) the duration of the P-wave (r = 0.45), (c) the duration of the QRS interval (r = 0.60), (d) the duration of the QT interval (r = 0.72), and the QTc ratio (r = 0.39). No statistically significant correlations were found between the amplitudes of the P- and R-waves and either body weight or age. We found that the QTc values of swine whose body weight was greater than 70 kg were significantly higher (*P* < 0.01) (Figure 2) than those of swine whose body weight was less than 70 kg. We also found that the heart’s electrical axis is unrelated to body weight or age. Typically, we found that values of the heart’s electrical axis ranged from 40° for swine weighing between 70–79 kg to 103° for swine weighing between 20–29 kg. The overall range of the heart’s electrical axis for all pigs was between 17-145°.

**Table 1:** **Summary of the results of the analysis of lead II electrocardiograms in healthy domestic swine according to their body weight in 10 kg increments**

**Body weight (kg)**	**P-wave amplitude (millivolts)**	**P-wave duration (seconds)**	**Duration of PQ interval (seconds)**	**Duration of QRS interval (seconds)**	**R-wave amplitude (millivolts)**	**Duration of QT interval (seconds)**	**QTc ratio (ms/√s)**	**Heart’s electrical axis (degrees)**	**Number of animals**
20-29	0.11 ± 0.02	0.06 ± 0.01	0.10 ± 0.02	0.07 ± 0.01	0.46 ± 0.25	0.26 ± 0.03	392 ± 33	103 ± 49	11
30-39	0.13 ± 0.06	0.06 ± 0.01	0.11 ± 0.02	0.07 ± 0.01	0.36 ± 0.16	0.27 ± 0.03	394 ± 25	80 ± 65	23
40-49	0.14 ± 0.03	0.06 ± 0.01	0.11 ± 0.02	0.07 ± 0.01	0.43 ± 0.25	0.28 ± 0.03	289 ± 39	91 ± 62	18
50-59	0.15 ± 0.04	0.07 ± 0.01	0.12 ± 0.02	0.07 ± 0.01	0.38 ± 0.28	0.28 ± 0.02	381 ± 31	93 ± 79	8
60-69	0.15 ± 0.04	0.07 ± 0.01	0.12 ± 0.02	0.07 ± 0.01	0.38 ± 0.20	0.28 ± 0.02	379 ± 44	87 ± 24	8
70-79	0.15 ± 0.03	0.07 ± 0.01	0.12 ± 0.02	0.07 ± 0.01	0.43 ± 0.20	0.29 ± 0.08	410 ± 23	45 ± 44	9
80-89	0.15 ± 0.03	0.07 ± 0.01	0.12 ± 0.01	0.07 ± 0.01	0.39 ± 0.20	0.31 ± 0.05	418 ± 39	96 ± 74	12
90-99	0.15 ± 0.04	0.07 ± 0.01	0.12 ± 0.01	0.07 ± 0.01	0.25 ± 0.35	0.33 ± 0.05	419 ± 33	56 ± 85	10
100-109	0.15 ± 0.05	0.07 ± 0.01	0.12 ± 0.02	0.07 ± 0.01	0.21 ± 0.12	0.32 ± 0.03	409 ± 46	86 ± 71	11
110-119	0.16 ± 0.05	0.08 ± 0.02	0.13 ± 0.02	0.08 ± 0.01	0.27 ± 0.19	0.33 ± 0.02	398 ± 40	70 ± 62	16
120-129	0.16 ± 0.06	0.08 ± 0.02	0.14 ± 0.02	0.08 ± 0.01	0.38 ± 0.32	0.35 ± 0.07	446 ± 36	78 ± 24	9
130-139	0.16 ± 0.04	0.08 ± 0.01	0.14 ± 0.02	0.08 ± 0.01	0.37 ± 0.24	0.35 ± 0.03	428 ± 33	91 ± 37	21
140-149	0.16 ± 0.04	0.08 ± 0.01	0.15 ± 0.02	0.08 ± 0.01	0.47 ± 0.36	0.36 ± 0.03	446 ± 33	62 ± 88	6
150-159	0.16 ± 0.05	0.08 ± 0.01	0.15 ± 0.03	0.09 ± 0.01	0.54 ± 0.18	0.38 ± 0.04	429 ± 28	77 ± 22	8

Values are displayed as mean ± standard deviation.

N = 170.

**Table 2:** **Summary of the results of the analysis of lead II electrocardiograms in healthy domestic swine according to their age**

**Age (months)**	**P-wave amplitude (millivolts)**	**P-wave duration (seconds)**	**Duration of PQ interval (seconds)**	**Duration of QRS interval (seconds)**	**R-wave amplitude (millivolts)**	**Duration QT interval (seconds)**	**QTc ratio (ms/√s)**	**Heart’s electrical axis (degrees)**	**Number of animals**
2	0.11 ± 0.02	0.059 ± 0.007	0.1 ± 0.02	0.07 ± 0.008	0.41 ± 0.23	0.26 ± 0.03	383 ± 39	91 ± 48	19
3	0.14 ± 0.04	0.060 ± 0.009	0.11 ± 0.01	0.07 ± 0.006	0.39 ± 0.19	0.27 ± 0.02	388 ± 34	86 ± 64	33
4	0.14 ± 0.04	0.065 ± 0.011	0.11 ± 0.02	0.07 ± 0.011	0.39 ± 0.21	0.27 ± 0.05	389 ± 31	82 ± 70	25
5	0.14 ± 0.03	0.068 ± 0.009	0.12 ± 0.01	0.07 ± 0.009	0.32 ± 0.25	0.32 ± 0.04	408 ± 26	85 ± 87	24
6	0.15 ± 0.05	0.070 ± 0.021	0.13 ± 0.02	0.08 ± 0.012	0.27 ± 0.22	0.33 ± 0.04	407 ± 45	87 ± 24	32
7	0.16 ± 0.04	0.070 ± 0.013	0.14 ± 0.01	0.08 ± 0.011	0.38 ± 0.26	0.35 ± 0.03	437 ± 36	84 ± 50	29
8	0.16 ± 0.04	0.080 ± 0.012	0.15 ± 0.02	0.09 ± 0.011	0.54 ± 0.18	0.38 ± 0.04	429 ± 39	60 ± 52	8

Values are displayed as mean ± standard deviation.

N = 170.

**Figure 3:**
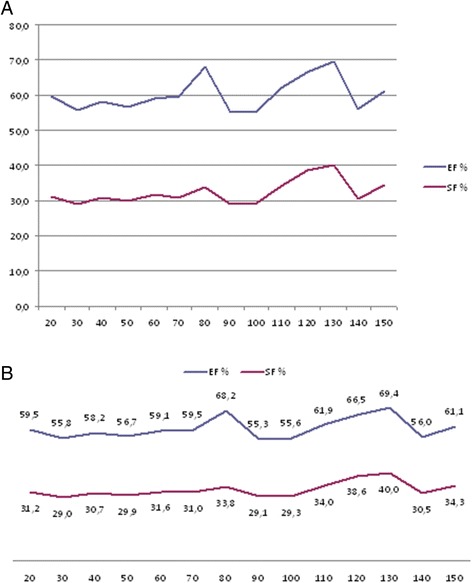
**End-diastolic and end-systolic diameters (cm) of the left ventricle (A) and fractional shortening (%), ejection fraction (%) of the left ventricle (B) of healthy domestic swine according to the body weight in 10 kg increments.** LVIDd - internal left ventricular dimensions at end-diastole; LVIDs - internal left ventricular dimensions at end-systole; EF – ejection fraction; SF – fractional shortening.

**Figure 4:**
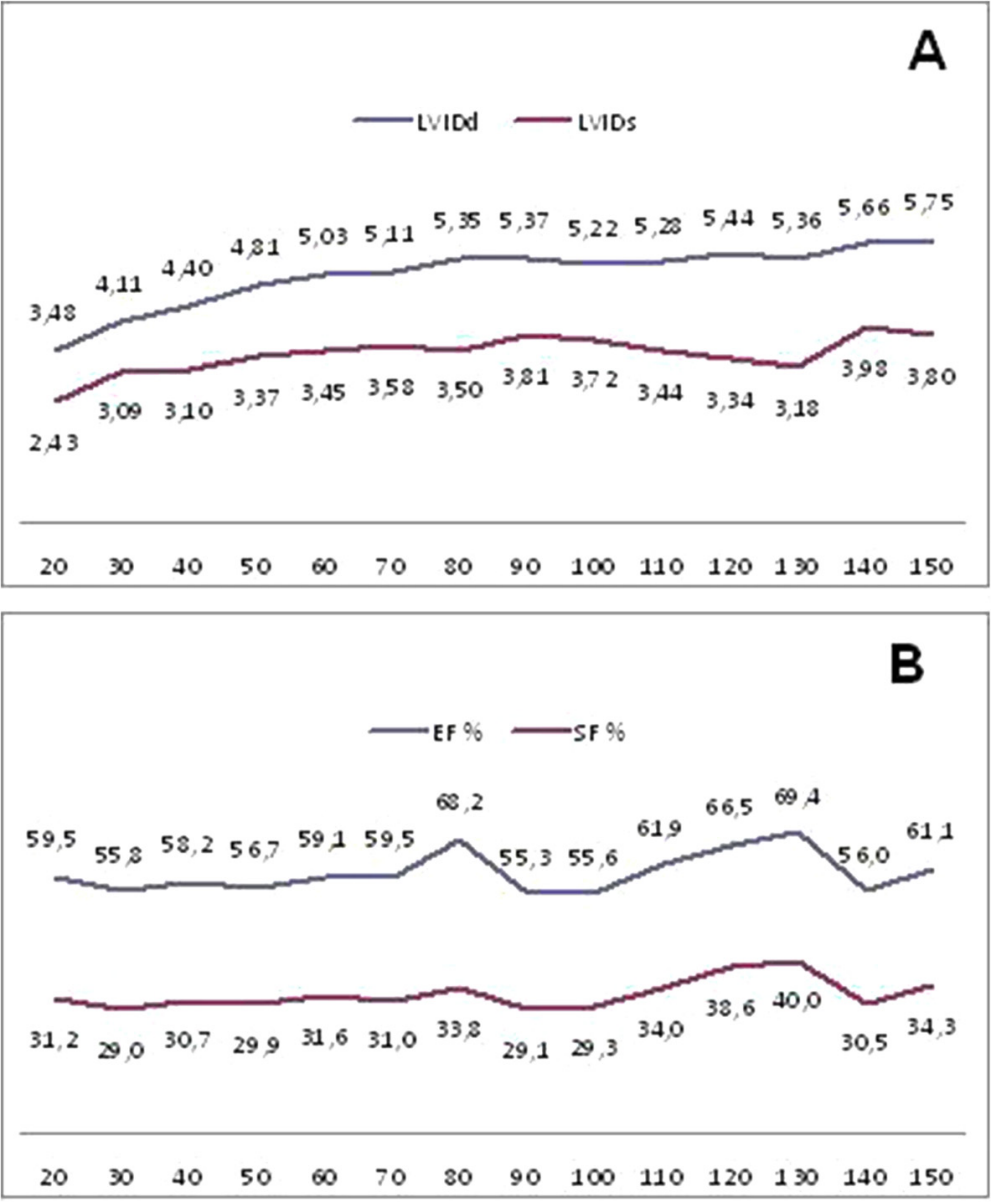
**Left ventricular end-diastolic and end-systolic diameters (cm) (A) and left ventricular shortening, ejection fractions (B) of healthy domestic swine according to their body weight.** LVIDd - internal left ventricular dimensions at end-diastole (cm); LVIDs - internal left ventricular dimensions at end-systole (cm); EF - ejection fraction (%); FS – fractional shortening (%).

**Figure 5:**
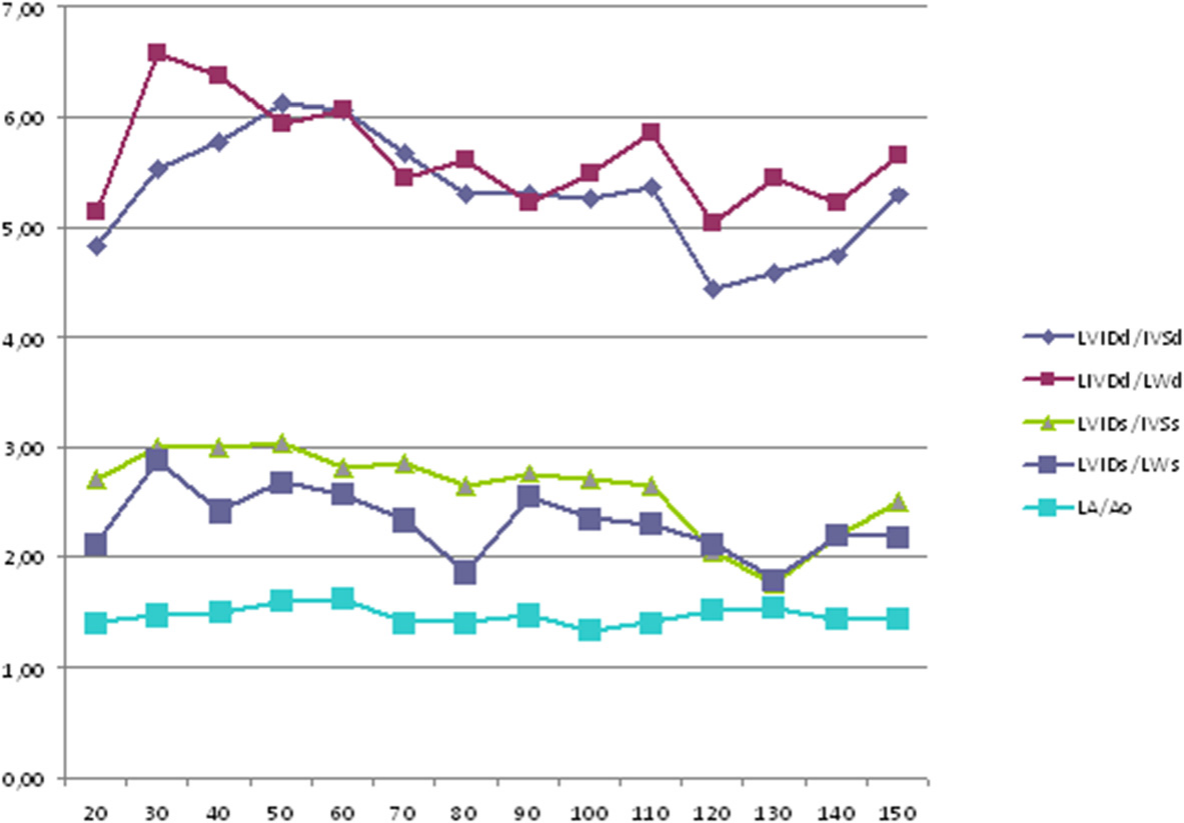
**Left ventricular internal diameter: wall thickness ratio and the left atrial-to-aortic root diameter ratio in healthy domestic swine according to their body weight in 10 kg increments.** LVIDd - internal left ventricular dimensions at end-diastole; IVSd - end-diastolic thickness of the interventricular septum; LVIDs - internal left ventricular dimensions at end-systole; IVSs - end-systolic thickness of the interventricular septum; LPWs – left ventricular posterior wall in end-systole; LA/Ao - left atrial-to-aortic root diameter ratio.

The results of the analysis of the echocardiograms are displayed in Tables 3, 4, 5, 6, 7 and 8 and in Figures 3, 4 and 5. We found that the thickness of the left ventricular wall and the internal diameter of the left ventricle increased with age and body weight. We also found a positive trend between body weight and EF (r = 0.165), as well as a statistically significant positive correlation (*P* < 0.05) between body weight and FS (r = 0.216), the left ventricular internal end-diastolic (r = 0.875), and the end-systolic diameter (r = 0.583) (Table 3 and Figure 3). We also found a positive correlation between age, body weight, and the ratio of the left ventricular diameter to its wall thickness, as well as the LA/Ao (Table 3 and Figures 4 and 5). No significant correlations were found between body weight and (a) the LA/Ao, whose sizes were between 1.4–1.59, (b) the RWT) of the left ventricle, whose thickness was between 0.32–0.39 cm, (c) the sum of the left ventricular wall thickness (defined as septal wall thickness plus posterior wall thickness), whose values were between 1.6–2.56 cm, (d) the relative interventricular septal thickness, whose thicknesses were between 0.86–1.2 cm, (e) the septal thickening fraction, whose values were between 20–36%, and (g) the fraction of left ventricular free wall thickening, whose values were between 36–43%. We found that body weight was positively correlated (*P* < 0.05) with the myocardial velocity during early systole (Sm1) (r = 0.334), the myocardial velocity during systole (Sm2) (r = 0.393), the RIVRT (r = 0.456), and the RIVRTp (r = 0.589), and these findings suggest that increasing body weight influences systolic function of the left ventricle. Since no positive correlations were found between body weight and the velocity of the Em wave (0.11-0.25 m/s), the velocity of the Am wave (0.08-0.18 m/s), and the Em/Am ratio (0.9-2.9), these findings indicate that increasing body weight has no effect on the diastolic function of the left ventricle.

**Table 3:** **Summary of the results of the analysis of the left ventricular echocardiograms in healthy domestic swine according to body weight in 10 kg increments**

**Body weight (kg)**	**End-diastole**			**End-systole**			**FS (%)**	**EF (%)**	**Number of animals**
	**IVSd (cm)**	**LVIDd (cm)**	**LPWd (cm)**	**IVSs (cm)**	**LVIDs (cm)**	**LPWs (cm)**			
20-29	0.73 ± 0.71	3.48 ± 0.31	0.67 ± 0.08	0.96 ± 0.22	2.43 ± 0.31	1.10 ± 0.18	29 ± 4	55 ± 7	11
30-39	0.77 ± 0.20	4.09 ± 0.32	0.69 ± 0.11	1.03 ± 0.09	3.06 ± 0.61	1.19 ± 0.16	31 ± 4	56 ± 6	23
40-49	0.78 ± 0.06	4.36 ± 0.34	0.71 ± 0.06	1.05 ± 0.14	3.07 ± 0.23	1.19 ± 0.16	31 ± 3	58 ± 5	18
50-59	0.79 ± 0.07	4.79 ± 0.44	0.82 ± 0.08	1.15 ± 0.12	3.42 ± 0.33	1.29 ± 0.16	30 ± 4	57 ± 6	8
60-69	0.84 ± 0.03	4.88 ± 0.31	0.92 ± 0.09	1.16 ± 0.11	3.42 ± 0.44	1.47 ± 0.14	32 ± 4	58 ± 5	8
70-79	0.89 ± 0.12	5.03 ± 0.26	0.95 ± 0.12	1.41 ± 0.12	3.53 ± 0.29	1.49 ± 0.23	33 ± 5	59 ± 7	9
80-89	0.98 ± 0.08	5.09 ± 0.43	0.95 ± 0.05	1.22 ± 0.21	3.44 ± 0.36	1.67 ± 0.25	33 ± 3	59 ± 6	12
90-99	0.98 ± 0.08	5.34 ± 0.24	0.96 ± 0.17	1.33 ± 0.23	3.68 ± 0.35	1.67 ± 0.16	30 ± 4	59 ± 7	10
100-109	0.98 ± 0.11	5.39 ± 0.28	0.98 ± 0.13	1.34 ± 0.11	3.72 ± 0.31	1.66 ± 0.22	31 ± 5	59 ± 7	11
110-119	1.04 ± 0.11	5.45 ± 0.54	1.07 ± 0.08	1.36 ± 0.16	3.72 ± 0.61	1.66 ± 0.13	32 ± 7	60 ± 9	16
120-129	1.14 ± 0.15	5.48 ± 0.2	1.08 ± 0.15	1.66 ± 0.28	3.69 ± 0.78	1.68 ± 0.35	33 ± 8	64 ± 14	9
130-139	1.16 ± 0.21	5.63 ± 0.51	1.06 ± 0.13	1.70 ± 0.35	3.69 ± 0.68	1.83 ± 0.25	34 ± 1	65 ± 10	21
140-149	1.12 ± 0.14	5.67 ± 0.41	1.08 ± 0.09	1.64 ± 0.23	3.87 ± 0.67	1.83 ± 0.26	30 ± 8	63 ± 13	6
150-160	1.06 ± 0.16	5.77 ± 0.46	1.06 ± 0.11	1.65 ± 0.20	4.11 ± 0.46	1.76 ± 0.18	32 ± 7	61 ± 9	8

IVS - interventricular septum thickness, LVID – left ventricular internal diameter, LPW – left posterior wall thickness, LPWs –left ventricular posterior wall in end-systole, FS – fractional shortening, EF – ejection fraction.

Values are displayed as mean ± standard deviation.

N = 170.

**Table 4:** **Summary of the results of the analysis of the left ventricular echocardiograms in healthy domestic swine according to age**

**Age (month)**	**End-diastole**			**End-systole**			**FS (%)**	**EF (%)**	**Number of animals**
	**IVSd (cm)**	**LVIDd (cm)**	**LPW (cm)**	**IVSs (cm)**	**LVIDs (cm)**	**LPWs (cm)**			
2	0.70 ± 0.08	3.70 ± 0.42	0.64 ± 0.11	0.99 ± 0.14	2.62 ± 0.34	1.12 ± 0.19	30 ± 4	58 ± 6	19
3	0.81 ± 0.15	4.27 ± 0.33	0.73 ± 0.09	1.06 ± 0.13	3.11 ± 0.51	1.17 ± 0.11	30 ± 4	57 ± 6	33
4	0.84 ± 0.09	4.93 ± 0.34	0.88 ± 0.11	1.17 ± 0.12	3.41 ± 0.37	1.40 ± 0.19	32 ± 4	59 ± 6	25
5	0.97 ± 0.08	5.25 ± 0.42	0.99 ± 0.12	1.29 ± 0.18	3.54 ± 0.42	1.67 ± 0.21	32 ± 5	62 ± 8	24
6	0.12 ± 0.14	5.44 ± 0.41	0.97 ± 0.15	1.44 ± 0.30	3.69 ± 0.58	1.59 ± 0.23	32 ± 8	60 ± 10	32
7	1.14 ± 0.19	5.61 ± 0.47	1.04 ± 0.12	1.65 ± 0.26	3.65 ± 0.72	1.83 ± 0.24	35 ± 8	62 ± 11	29
8	1.11 ± 0.16	5.88 ± 0.46	1.04 ± 0.11	1.58 ± 0.27	3.99 ± 0.70	1.76 ± 0.18	33 ± 7	59 ± 10	8

IVS - interventricular septum thickness, LVID – left ventricular internal diameter, LPW – left posterior wall thickness, LPWs –left ventricular posterior wall in end-systole, FS – fractional shortening, EF – ejection fraction.

Values are displayed as mean ± standard deviation.

N = 170.

**Table 5:** **Echocardiographic measurements of cardiac dimensions of healthy domestic swine according to their body weight**

**Body weight (kg)**	**LA/Ao**	**RWT**	**LWd + LWs (cm)**	**IVSd/LWd**	**ST IVS (%)**	**ST LPW (%)**	**Number of animals**
20-29	1.40 ± 0.25	0.38	2.19	1.20	24	39	11
30-39	1.47 ± 0.20	0.34	1.77	1.21	25	42	23
40-49	1.49 ± 0.17	0.32	1.61	1.10	26	40	18
50-59	1.57 ± 0.27	0.34	1.75	0.95	31	36	8
60-69	1.58 ± 0.14	0.38	1.88	0.86	28	37	8
70-79	1.57 ± 0.16	0.38	2.08	0.94	36	36	9
80-89	1.59 ± 0.14	0.37	2.06	1.06	20	43	12
90-99	1.53 ± 0.25	0.36	2.19	0.94	26	43	10
100-109	1.52 ± 0.14	0.36	2.20	0.98	27	41	11
110-119	1.49 ± 0.21	0.39	2.30	1.00	24	36	16
120-129	1.49 ± 0.20	0.39	2.52	1.05	31	36	9
130-139	1.47 ± 0.17	0.38	2.56	1.15	32	42	21
140-149	1.49 ± 0.19	0.38	2.43	1.08	32	41	6
150-160	1.46 ± 0.23	0.37	2.39	1.04	36	40	8

LA/Ao - left atrial size-to-aortic root diameter ratio; RWT - relative wall thickness; LWd + LWs - sum of maximum left ventricular wall thickness, IVSd/LWd - relative interventricular septal thickness; ST IVS - interventricular septal systolic thickening; ST LW left ventricular free wall thickening. Values are displayed as mean ± standard deviation.

N =170.

**Table 6:** **Echocardiographic measurements of cardiac dimensions of healthy domestic swine according to their age**

**Age (month)**	**LA/Ao**	**RWT**	**LWd + LWs (cm)**	**IVSd/LWd**	**ST IVS (%)**	**ST LPW (%)**	**Number of animals**
2	1.40 ± 0.21	0.34	1.76	1.34	29	43	19
3	1.51 ± 0.22	0.30	1.87	1.51	24	40	33
4	1.55 ± 0.20	0.36	2.28	2.43	28	37	25
5	1.62 ± 0.15	0.38	2.66	1.96	25	41	24
6	1.51 ± 0.22	0.36	2.56	2.01	28	39	32
7	1.45 ± 0.17	0.37	2.87	2.18	31	43	29
8	1.50 ± 0.21	0.35	2.81	2.08	34	41	8

LA/Ao - left atrial size-to-aortic root diameter ratio; RWT - relative wall thickness; LWd + LWs - sum of maximum left ventricular wall thickness; IVSd/LWd - relative interventricular septal thickness; ST IVS - interventricular septal systolic thickening; ST LW - left ventricular free wall thickening. Values are displayed as mean ± standard deviation.

N = 170.

**Table 7:** **Tissue Doppler parameters of the left ventricular free wall basal segment in healthy domestic swine according to their body weight**

**Body weight (kg)**	**Sm1 (meter/seconds)**	**Sm2 (meter/seconds)**	**Em (meter/seconds)**	**Am (meter/seconds)**	**Em/Am**	**RIVRT (milliseconds)**	**RIVRTp (milliseconds)**	**Number of animals**
20-29	0.09 ± 0.05	0.08 ± 0.01	0.18 ± 0.04	0.10 ± 0.03	2.0 ± 0.56	52 ± 20	142 ± 22	11
30-39	0.06 ± 0.02	0.80 ± 0.01	0.17 ± 0.04	0.10 ± 0.03	1.8 ± 0.04	48 ± 16	140.5 ± 16	23
40-49	0.08 ± 0.03	0.09 ± 0.01	0.17 ± 0.04	0.12 ± 0.04	1.6 ± 0.50	55 ± 11	151 ± 17	18
50-59	0.07 ± 0.02	0.09 ± 0.01	0.17 ± 0.03	0.11 ± 0.04	1.7 ± 0.43	55 ± 12	171 ± 13	8
60-69	0.09 ± 0.02	0.11 ± 0.02	0.17 ± 0.03	0.15 ± 0.05	1.3 ± 0.44	43 ± 15	152 ± 24	8
70-79	0.09 ± 0.03	0.11 ± 0.01	0.19 ± 0.03	0.12 ± 0.03	1.6 ± 0.44	45 ± 21	175 ± 27	9
80-89	0.09 ± 0.03	0.10 ± 0.03	0.19 ± 0.02	0.13 ± 0.04	1.8 ± 0.47	49 ± 14	159 ± 22	12
90-99	0.10 ± 0.03	0.11 ± 0.02	0.16 ± 0.03	0.13 ± 0.03	1.4 ± 0.28	50 ± 16	167 ± 27	10
100-109	0.11 ± 0.04	0.10 ± 0.02	0.19 ± 0.02	0.12 ± 0.03	1.7 ± 0.47	70 ± 29	171 ± 46	11
110-119	0.10 ± 0.03	0.11 ± 0.02	0.20 ± 0.02	0.11 ± 0.03	1.9 ± 0.47	71 ± 24	179 ± 35	16
120-129	0.10 ± 0.04	0.09 ± 0.01	0.17 ± 0.03	0.1 ± 0.02	1.7 ± 0.24	87 ± 21	189 ± 21	9
130-139	0.10 ± 0.03	0.10 ± 0.01	0.17 ± 0.04	0.11 ± 0.03	1.6 ± 0.65	85 ± 63	181 ± 22	21
140-149	0.10 ± 0.04	0.11 ± 0.02	0.16 ± 0.03	0.11 ± 0.04	1.6 ± 0.55	86 ± 24	183 ± 25	6
150-160	0.10 ± 0.04	0.10 ± 0.02	0.18 ± 0.02	0.13 ± 0.04	1.4 ± 0.31	164 ± 17	186 ± 1	8

Waves: Sm1 – early systolic, Sm2 – systolic, Em – early diastolic, Am – late diastolic; RIVRT – isovolumic relaxation time, RIVRTp – time from the onset of QRS complex to the peak of the Sm2 wave.

Values are displayed as mean ± standard deviation.

N = 170.

**Table 8:** **Tissue Doppler Imaging parameters of the left ventricular free wall basal segment in healthy domestic swine related to their age**

**Age (months)**	**Sm1 (meter/seconds)**	**Sm2 (meter/seconds)**	**Em (meter/seconds)**	**Am (meter/seconds)**	**Em/Am**	**RIVRT (milliseconds)**	**RIVRTp (milliseconds)**	**Number of animals**
2	0.08 ± 0.05	0.07 ± 0.01	0.18 ± 0.04	0.09 ± 0.03	2.0 ± 0.51	50 ± 17	140 ± 21	19
3	0.07 ± 0.03	0.08 ± 0.01	0.17 ± 0.04	0.11 ± 0.03	1.8 ± 0.04	54 ± 17	148 ± 16	33
4	0.08 ± 0.03	0.10 ± 0.01	0.17 ± 0.04	0.13 ± 0.04	1.5 ± 0.52	49 ± 18	167 ± 25	25
5	0.09 ± 003	0.11 ± 0.02	0.17 ± 0.03	0.13 ± 0.04	1.6 ± 0.45	50 ± 14	159 ± 34	24
6	0.11 ± 0.03	0.10 ± 0.02	0.17 ± 0.03	0.11 ± 0.03	1.8 ± 0.44	75 ± 25	183 ± 27	32
7	0.10 ± 0.03	0.10 ± 0.01	019 ± 0.03	0.11 ± 0.03	1.6 ± 0.62	86 ± 54	182 ± 23	29
8	0.12 ± 0.04	0.11 ± 0.03	0.19 ± 0.02	0.13 ± 0.04	1.5 ± 0.30	65 ± 23	164 ± 16	8

Waves: Sm1 – early systolic, Sm2 – systolic, Em – early diastolic, Am – late diastolic; RIVRT – isovolumic relaxation time, RIVRTp – time from the onset of QRS complex to the peak of the Sm2 wave.

Values are displayed as mean ± standard deviation.

N = 170.

## Discussion

In this investigation, we found that HR is strongly correlated with the body weight of healthy domestic swine. Specifically, we found that the average HR for swine with a body weight less than 70 kg is 100 beats/minute (bpm). For swine with a body weight greater than 70 kg, we found that the average HR was below 90 bpm and was even less than 80 bpm in swine with a body weight of more than 120 kg. Activation of the sympathetic system before drug administration and the mechanism of action of any administered drug should be considered when interpreting the ECG recordings and HR measurements of an anaesthetised animal. Experience has taught us that 20 minutes is usually required for stabilisation of the cardiovascular system after anaesthetising an animal and before collecting any recordings of cardiovascular function. During this 20-minute stabilisation period, large fluctuations in HR, blood pressure, and some echocardiographic measures are frequently detected due to different activity levels of the sympathetic nervous system before anaesthetic premedication and the rate of drug distribution after premedication and the induction of anaesthesia.

We found that body weight of the swine is positively correlated with the duration of the P-wave, the duration of the QRS interval, the duration of the QT interval, and the QTc ratio. We also found that pig’s heart weight increases as its body weight increases, a finding which is confirmed in the echocardiograms and this correlation underlies the prolonged conduction time that we found in the heavy pigs. We did not find any significant correlations between body weight and age and the amplitudes of the P- and R-waves. In fact, the amplitudes of the P-wave are comparable at all weights and ages. We also did not find any positive correlation between the amplitude of the R-wave and the configuration of the QRS complex, whose configuration is a characteristic feature of its ECG and differs from that found in other species. Specifically, the configuration of the QRS complex in the lead II ECG appears to have two forms, a qRs (normal amplitude R-wave) form and a qrS (small-amplitude or small r-wave) form [18]. These differences in the configurations of the QRS complex are principally due to differences in pathways of ventricular activation. Probably the cause of this situation is left anterior hemiblock of the left bundle branch in the left ventricle. This block may be congenital in swine (breeding, exterior feature selection, inbreeding) and the electrical activation of the left ventricle run across right to left side and from posterior to anterior part of the septum. Dukes and Szabuniewicz [19] almost 50 years ago proposed an explanation that this small r-wave pattern derives from the anastomotic branches between the left and right main bundles and that probably underpin polymorphic the ventricular tachycardia and ventricular fibrillations - arrhythmias that have been described in swine but in our opinion it could not withstand the test of time today. We found small r-waves in the lead II ECG in about 60% of the investigated swine and normal R-waves in 40% of the investigated swine. We presume that this finding is the reason why we did not find a correlation between the amplitude of the R- wave, age, LV dimensions and body weight. This will be the subject of a detailed further research by our group in the future.

**Figure 6:**
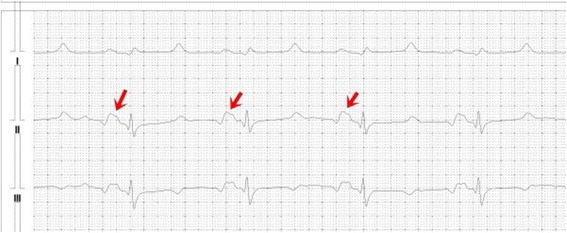
**A typical triphasic P-wave in the lead II and III electrocardiogram in a healthy domestic swine.** Heart Rate = 71 beats/minute, Speed 50 mm/second, Amplitude 1 mV = 10 mm. Red arrows shows triphasic P-wave.

We also found the other characteristic feature that distinguishes the swine’s ECG from that of other species: a biphasic, triphasic or multiphasic P-wave with an initial negative phase in the lead II electrocardiogram in 50% of the investigated swine, regardless of their body weight (Figure 6). In our investigation, the ECG was recorded when the pig was lying in a left lateral position. We found that the morphology of the pig’s P-wave is not dependent on the pig’s position during the recording of ECG. This finding is consistent with that described by Dukes and Szabuniewicz [19] who described a similar P-wave morphology when the ECG was recorded when the pig is lying in a sternal position.

We also detected variable and different morphologies of the P-wave and fading of the negative phase in the ECGs from other leads that were recorded in the same animal. The duration and dispersion of the P-wave are considered to reflect the electrophysiological properties of atrial muscles. Since the electrical activity of cardiac muscle, as depicted in an ECG, is strictly related to conduction in specific atrial areas, regional depolarization disorders may underpin the variation in the duration and morphology of the P-wave in the various leads [20]. For example, the changes in the duration and morphology of the P-wave may depict intra- and interatrial conduction disorders as well as non-homogenous propagation of the impulse from the sinoatrial node in swine [21].

A prolonged duration of the QT interval is another characteristic feature of the swine’s ECG, and increased sensitivity to arrhythmia induction, such as ventricular fibrillation and polymorphic ventricular tachycardia, has been attributed to prolonged ventricular repolarization [22]. Although a prolonged duration of the QT interval can be a trigger for an arrhythmia, it is not arrhythmogenic *per se* [23]. We found that the QTc ratio tended to lengthen in 6-month-old swine with a body weight greater than 70 kg. We surmise that this tendency towards an elongated QTc and biphasic T-wave may be a reflection of repolarization heterogeneity. Long depolarization and repolarization in pigs is associated with a high risk for SCD [24]. Alsheikh-Ali and colleagues [4] concluded that the susceptibility to SCD might be due to abnormalities in cardiac depolarization and repolarization, such as having a QTc ratio longer than 460 milliseconds.

We found a positive correlation between body weight and the echocardiographic measures of the size and function of the left ventricle, a correlation which was also previously reported in young swine by Gwathmey *et al.* [13]. Specifically, we found that heart size proportionally increases with increasing body weight, and of the echocardiographic measures of cardiac dimensions, we found that the increase in the left atrium is the smallest. We also found interesting cyclic changes in FS and EF of the investigated swine but we are unable to provide satisfactory explanations for their genesis and importance. Specifically, we found that the values of FS declined by about 30% in pigs whose body weight had increased by 20, 40, and 60 kg. This result suggests that the length of the cardiac cycle, in which FS and EF declines, increases with increases in body weight. We also found that the smallest FS occurred in swine with body weights of 25, 55, 95, and 145 kg. None of these changes were statistically significant because of individual differences between swine and the relatively small number of animals in each of the study groups (about ten swine per group). This phenomenon requires further investigation in order to assess its clinical significance and to determine whether it is linked to SCD, to which swine are predisposed. Such fluctuations were not observed when the animals were assigned to age groups.

Corya and colleagues [25] reported that the values of ST IVS% and ST LPW% in swine with a body weight greater than 65 kg are the same as those of humans. We also calculated the ratio of the left ventricular end-systolic and end-diastolic diameters to the interventricular septal and free wall thickness in order to determine changes in left ventricular dimensions. We found no changes in the ventricular dimensions of the investigated swine. The left ventricular RWT is also a measure of left ventricular dimensions and this measure is frequently used to diagnose left ventricular dilation and hypertrophy [26]. We found that its value in our swine is 0.32–0.39 cm, and that this value is modestly linked to increasing body weight.

We found that none of the echocardiographic measures were influenced by the pig’s sex. Sex differences in echocardiographic parameters occur in humans, and these differences seem to be due to the different body weights of men and women. We found that the rates of increase in body weight and cardiac weights in the pigs were the same and were not sex-dependent. Shapiro [27] reported that posterior wall and septal thickness and left ventricular mass could be used to diagnose hypertrophic cardiomyopathy and asymmetrical left ventricular hypertrophy in humans. Accordingly, we calculated the left ventricular wall thickness in end-diastole (IVSd + LWd) in the pigs. We found that the thickness did not exceed 1.2 cm and that thickness was not related to either the body weight or sex of the pig. We also found that left ventricular wall thickness of pigs weighing 30–110 kg was the same as that which has been reported in humans [28,29].

TDI is a relatively new ultrasound technique and is used to measure the velocity of myocardial motion and evaluate regional myocardial function. In humans, TDI has been reported to be useful for predicting the prognosis of symptomatic and asymptomatic patients with hypertrophic cardiomyopathy [26]. Matsumura and colleagues [30] have reported that the mitral annular velocities in humans and swine are similar. In this investigation, we measured the circular fibre strain rate in the basal segment of the left ventricular free wall in the study pigs, and the longitudinal fibre strain rates in a few pigs. We also measured basal segment motion in the apical two-chamber view (data not shown) and we found that the results were very similar to those measurements that were found in the parasternal short-axis view. We were unable to measure mitral annular motion in all swine because good quality images were not obtained in the transthoracic echocardiogram when the swine were very large. Accordingly, a transoesophageal probe should be used to obtain this information in swine with a body weight greater than 130 kg. Images of parasternal short-axis views and apical four-chamber views were obtained in all swine, the quality of the images of apical four-chamber views in swine with a body weight greater than 130 kg was low. Hence, such images have no diagnostic value.

In conclusion, we found that many electro- and echocardiographic measures of cardiac morphology and function of healthy domestic swine are related to body weight. When the electro- and echocardiographic measures of domestic swine and humans are compared, the most comparable electrocardiographic values are those that were determined in swine whose body weights were less than 70 kg. In contrast, the most comparable echocardiographic values are those that were determined in swine with a body weight between 40–110 kg.
